# Modeling and simulation of high energy density lithium-ion battery for multiple fault detection

**DOI:** 10.1038/s41598-022-13771-4

**Published:** 2022-06-13

**Authors:** Chandrani Sadhukhan, Swarup Kumar Mitra, Suvanjan Bhattacharyya, Eydhah Almatrafi, Bahaa Saleh, Mrinal Kanti Naskar

**Affiliations:** 1 Electrical Engineering Department, MCKV Institute of Engineering, Liluah, Howrah, West Bengal 712104 India; 2 Electronic & Telecommunication Engineering Department, MCKV Institute of Engineering, Liluah, Howrah, West Bengal 712104 India; 3 Department of Mechanical Engineering, Birla Institute of Technology & Science, Pilani, Pilani Campus, VidyaVihar, Rajasthan 333 031 India; 4 Mechanical Engineering Department, College of Engineering Rabigh, King Abdulaziz University, Jeddah, Saudi Arabia; 5 K. A. CARE Energy Research and Innovation Centre, King Abdulaziz University, Jeddah, 21589 Saudi Arabia; 6 Center of Excellence in Renewable Energy and Power System, King Abdulaziz University, Jeddah, Saudi Arabia; 7 Mechanical Engineering Department, College of Engineering, Taif University, P.O. Box 11099, Taif, 21944 Saudi Arabia; 8 Electronic & Telecommunication Engineering Department, Jadavpur University, Jadavpur, West Bengal 700032 India

**Keywords:** Fuel cells, Renewable energy, Electrical and electronic engineering, Energy infrastructure, Mechanical engineering

## Abstract

Lithium-ion battery, a high energy density storage device has extensive applications in electrical and electronic gadgets, computers, hybrid electric vehicles, and electric vehicles. This paper presents multiple fault detection of lithium-ion battery using two non-linear Kalman filters. A discrete non-linear mathematical model of lithium ion battery has been developed and Unscented Kalman filter (UKF) is employed to estimate the model parameter. Occurrences of multiple faults such as over-charge, over-discharge and short circuit faults between inter cell power batteries, affects the parameter variation of system model. Parallel combinations of some UKF (bank of filters) compare the model parameter variation between the normal and faulty situation and generates residual signal indicating different fault. Simulation results of multiple numbers of statistical tests have been performed for residual based fault diagnosis and threshold calculation. The performance of UKF is then compared with Extended Kalman filter (EKF) with same battery model and fault scenario. The simulation result proves that UKF model responses better and quicker than that of EKF for fault diagnosis.

## Introduction

The battery, an energy source has been used by the mankind since its invention more than two hundred years ago. After lots of developments, now-a-days batteries available are lighter in weight, higher energy storage capacity, enhanced safety features, and longer durability and found suitability in wide range of consumer and industrial applications^1,2^. Lithium batteries have been modified into lithium ion to make it rechargeable and applied in electrical gadgets, computers, hybrid electric vehicles, and electric vehicles etc. Considering the aspects like reliability and safety of electric vehicles, it is important to monitor the states of lithium ion cells during operation. This can be managed by collection of required data and subsequent estimation of states of cells through a battery management system (BMS)^3,4^. The performance of battery cell depends on current, voltage and temperature, and the state of cells include state of charge (SOC)^5–7^, state of health (SOH)^8–10^ and state of energy (SOE)^11^ and remaining useful life time (RUL)^12,13^. The faults in electrical vehicle are indicated as (a) overcharge, (b) over-discharge (c) internal and external short circuit. The battery internal and external short circuit fault results in generation of huge amount of heat which induces thermal runway. Unchecked faults in the battery are irreversible in nature and may lead to damages when it is severe^14,15^. In order to nullify such situations, it is important to diagnose fault of the battery quickly and accurately. It has been observed from the literatures that diagnosis of fault of lithium ion battery is of growing interest among researchers both in industry and academic field. The efforts have been put by the researchers aiming to detect different battery faults using advanced methodologies and techniques. One such technique is observer-based fault diagnosis which offers improved robustness because of its capability to avoid battery fault information loss. That may be accomplished due to unknown disturbances and faulty initial condition. The inherent advantages of lower cost and high flexibility make the model based fault diagnosis techniques a viable solution for accurate fault diagnosis^16^. The Luenberger observer (LO) using a series of reduced order observers^17^ can be applied on battery-pack for fault detection. Some researchers proposed model-based short circuit fault analysis using advanced techniques like indentation^18^, nail penetration^19^, fabrication with defect structures^20^ and thermal runaway at extreme high temperatures^21^. In another model, output voltages and the actual output voltages of batteries can be compared during the EV operation process and the alarm system will be triggered when the absolute value of the voltage difference exceeds the threshold^22,23^. Also, Kalman filter finds its effective application for diagnosis of the fault in lithium-ion batteries^24,25^ in particular when optimal filter exhibits strong robustness with noisy signal. The model based fault detection methods facilitated with very high robustness can be used to detect faults of battery accurately. Adaptive Kalman filter based fault diagnosis for lithium ion battery is under consideration by many researchers^26–28^. Adaptive Kalman filter can estimate states of battery parameter by the process and measurement noise covariance adjusting which is not possible in case of Extended Kalman filter where information on noise statistics are considered to be the pre-requisite for proper functioning of the filter otherwise it may lead to inaccurate results. Recently overcharge and overdischarge of battery fault is discussed^29^. A review paper on Fault Mechanisms, Fault Features, and Diagnosis Procedures are discussed^30^.

Considering the wide application of lithium-ion batteries in various devices, it is desirable to manufacture batteries which will have higher energy density, power density and service life. The failure due to over-charge, over-discharge, short circuit between inter cells of lithium-ion battery could lead to performance degradation and system fault which in turn may cause inconvenience, faster aging and higher cost of maintenance, thermal runaway or even explosion. Therefore, it is imperative to design a reliable and robust battery management system for early detection of the faults of the battery during service condition. The overall performance is greatly dependent on critical functions such as State-of-Charge (SOC) and State-of-Health (SOH) estimations, over-charge, and under-charge protections etc. From the practical point of view, estimation of three faults, namely over-charge, over-discharge and short circuit fault between inter cell power of lithium-ion batteries will certainly improve the reliability and efficiency of the devices, gadgets, electric and hybrid electric vehicles etc.

It has been found that some published research papers concentrate only internal short circuit fault^18–20^ of the battery pack and some other works describes fault such as over-charge, over-discharge etc. No researcher has considered all these faults simultaneously of lithium-ion battery in their work using model based method. Most of the researchers have concentrated model based method using a single technique that is residual evaluation for estimation of the faults of the batteries^22,24–26^. The novelty of present work is to model based fault detection occurs on lithium-ion battery pack for over-charge, over discharge and short circuit fault between inter cell power of lithium-ion batteries simultaneously. In the present study, a systematic model based fault detection scheme is proposed using a bank of Unscented Kalman filter (UKF) on lithium ion battery pack model for multiple fault detection such as over-charge, over-discharge and short circuit fault between inter cell power of lithium-ion batteries. A statistical test has been performed for residual based fault diagnosis and threshold calculation. The performance of UKF then compared with bank of Extended Kalman filter (EKF) on same battery model with same fault scenario. Depending on battery usage, different model of battery such as experimental, empirical, electrochemical are used. The battery model is considered as an extension of the The venin model where over-charged, over-discharged and short circuit fault between inter cell power of lithium-ion batteries are taken as fault parameter. The proposed work is divided into two parts: (a) experimental (b) simulation. In experimental part battery cells are monitored offline for long time interval in case of over-charging and over-discharging and parameter variation due to over-charging, over-discharging are measured. A 123 26650 LiFePO_4_ battery (3.3 Volts, 2.5 Ah) cell was used in the experiment. Electrochemical impedance spectroscopy (EIS) technique is used to extract the circuit parameter variation during overcharging and discharging of the battery which is reflected in Tables 2 and 3. The parameter variations are incorporated in the battery model during simulation and run by two bank of filter such as UKF and EKF. The lithium ion battery states are estimated and also residual signal is generated by comparing estimated and measured output for each individual power cell using UKF bank. It has been shown that the UKF based fault diagnosis proves significant result when compared with EKF based approach.

## Proposed fault diagnosis scheme on battery pack using UKF/EKF bank

**Figure 1:**
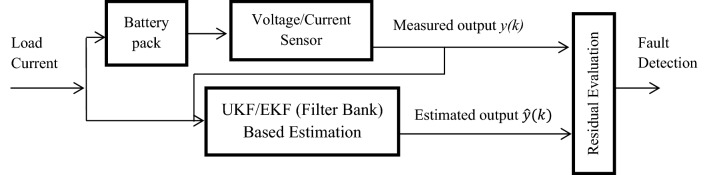
Schematic diagram of UKF/EKF bank-based fault detection scheme.

A model-based fault detection scheme for a battery pack using bank of UKF or EKF is represented in Fig. 1. To diagnose the fault due to overcharge, over discharge or short circuit fault in a battery pack, a bank of UKF or EKF works in parallel with the system. A series of voltage and current sensors are connected to the battery pack to measure voltage and current in each cell of battery pack. The various parameters, states of battery model can be measured by sensor provided data. The state space model of equivalent battery pack is designed and UKF or EKF banks are processed to get the estimated states of the system. The estimated data from filter and sensor provided data are compared and residual signal is generated. The mean of residual signal indicate the existence of fault in the system.

### Residual signal generation

The discrete state space model of any non-linear time invariant system (with fault) can be expressed as

1 $$x ({k + 1} ) = f ({x (k ), u (k)} ) + {F_{T}} (k ),$$

2 $$y\left(k\right)=g\left(x\left(k\right),u\left(k\right)\right) ,$$

where, *x(k), u(k)* and *y(k)* denotes the state vector, input signal and system output vector respectively at time step *k*. Nonlinear functions *f()* and *g()* are continuously differentiable with respect to time and *F*_*T*_*(k)* implies the occurrences of fault at time step *k.*

The discrete state space model of nonlinear Kalman Filter is given by

3 $$\widehat{x}\left(k+1\right)=f\left(\widehat{x}\left(k\right), u\left(k\right)\right)+w\left(k\right),$$

4 $$\widehat{y}\left(k\right)=g\left(\widehat{x}\left(k\right), u\left(k\right)\right)+v\left(k\right),$$

where, $\widehat{x}\left(k\right)$ and $\widehat{y}\left(k\right)$ denotes estimated state vector and estimated output vector of the filter at time step *k* respectively. Where *w(k)* and *v(k)* are independent zero mean Gaussian process and measurement noise. The process noise variance *Q*_*k*_ and measurement noise variance *R*_*k*_ are expressed as.

5 $$E\left[{w}_{i}{w}_{j}^{T}\right]= \left\{\begin{array}{l}{Q}_{k}\quad i=j\\ 0 \quad i\ne j\end{array}\right. ,$$

6 $$E\left[{v}_{i}{v}_{j}^{T}\right]= \left\{\begin{array}{l}{R}_{k}\quad i=j\\ 0 \quad i\ne j\end{array}\right..$$

From Eq. (2) and Eq. (4), the residual signal is expressed as

7 $$\begin{aligned} r_{d} = & \,y\left( k \right) - \hat{y}\left( k \right) \\ = & \,F\left( {w\left( k \right), v\left( k \right)} \right) + F_{T} \left( k \right), \\ \end{aligned}$$

where *F( )* is function of process *w(k)* and measurement noise *v(k)* sequence.

8 $$r_{d} = \left\{ {\begin{array}{*{20}l} {F\left( {w\left( k \right),~v\left( k \right)} \right)} \hfill & {\left( {healthy~\,as\,F_{T} \left( k \right) = 0~} \right)} \hfill \\ {F\left( {w\left( k \right),~v\left( k \right)} \right) + F_{T} \left( k \right)} \hfill & {\left( {faulty\,as\,F_{T} \left( k \right)~ \ne 0} \right)} \hfill \\ \end{array} .} \right.$$

If there exist any fault in the system ${F}_{T}\left(k\right)$), the filter output indicates the non-zero mean (NZM) residual sequences which is the summation of Gaussian noise and existing fault as given in Eq. (7). Simultaneous occurrences of multiple faults in the system each state of the filter output is indicated by NZM residual sequences.

**Figure 2:**
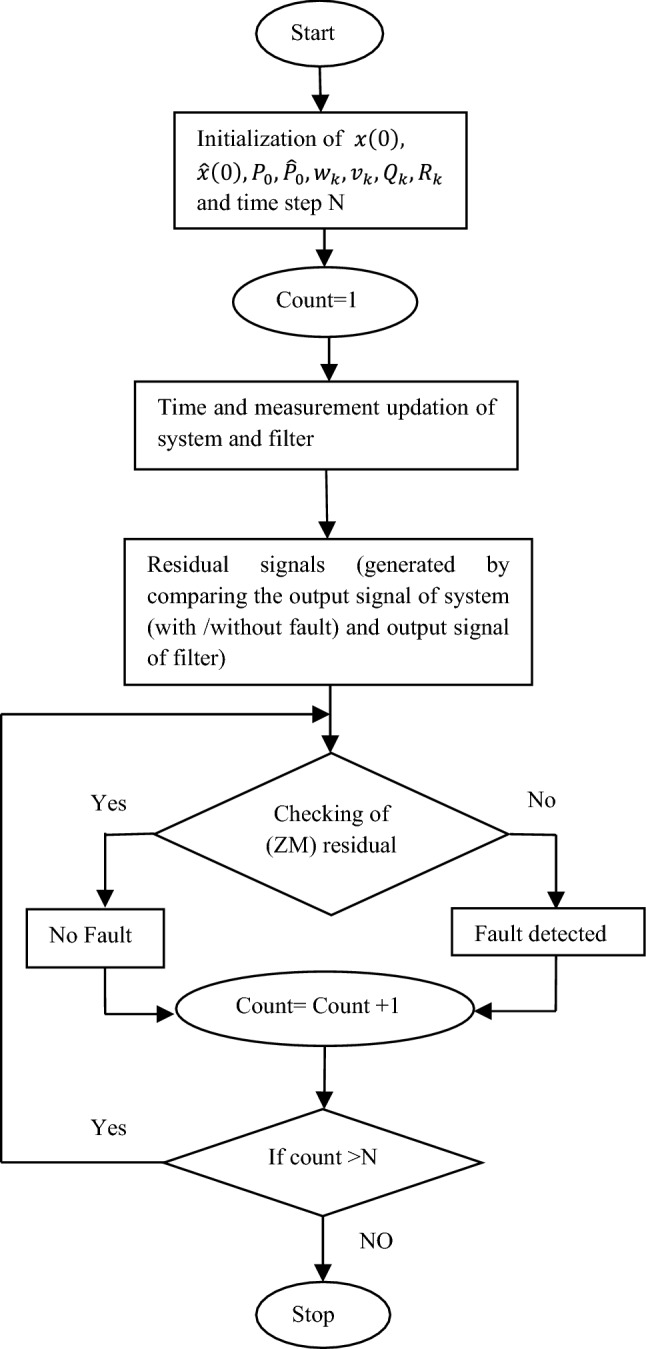
Residual based multiple fault diagnosis algorithm using UKF/EKF.

A multiple fault diagnosis scheme is explained in the flowchart as shown in Fig. 2. When a system is affected by n number of different faults such as ${F}_{T1}, {F}_{T2}, \dots .. {F}_{Tn}$, a bank of filters are utilized by incorporating each fault separately. The discrete state equation of each filter is represented as:

9 $$\left.\begin{array}{c}{\widehat{x}}_{1}\left(k+1\right)=f\left({\widehat{x}}_{1}\left(k\right), u\left(k\right)\right)+ {F}_{T1}\\ {\widehat{x}}_{2}\left(k+1\right)=f\left({\widehat{x}}_{2}\left(k\right), u\left(k\right)\right)+ {F}_{T2}\\ .\\ .\\ .\\ {\widehat{x}}_{n}\left(k+1\right)=f\left({\widehat{x}}_{n}\left(k\right), u\left(k\right)\right)+ {F}_{Tn}\end{array}\right\}.$$

The output equation of each filter are described by

10 $$\left.\begin{array}{c}\begin{array}{c}{\widehat{y}}_{1}\left(k\right)=g\left({\widehat{x}}_{1}\left(k\right), u\left(k\right)\right)\\ {\widehat{y}}_{2}\left(k\right)=g\left({\widehat{x}}_{2}\left(k\right), u\left(k\right)\right)\end{array}\\ .\\ .\\ .\\ .\\ {\widehat{y}}_{n}\left(k\right)=g\left({\widehat{x}}_{n}\left(k\right), u\left(k\right)\right)\end{array}\right\}.$$

The residual of each filter is the difference between the system output and the filtered output.

Residual of each filter are expressed as

11 $$\left.\begin{array}{c}{r}_{d1}={y}_{1}\left(k\right)- {\widehat{y}}_{1}(k) \\ {r}_{d2}={y}_{2}(k)- {\widehat{y}}_{2}(k)\\ .\\ .\\ .\\ {r}_{dn}={y}_{n}(k)- {\widehat{y}}_{n}(k)\end{array}\right\} .$$

The summary of UKF algorithm is given in Table 1. Residual based multiple fault diagnosis using UKF/EKF is shown in the flowchart given in Fig. 2. For *i* no. of cells are monitored by voltage or current sensor, if any fault occurs, the estimated state of filter output will not match with the sensor output data as a result Non Zero Mean (NZM) residual signal obtained. When fault does not occur in the system it shows output as Zero Mean (ZM) residual of process and measurement noise.

**Table 1:**
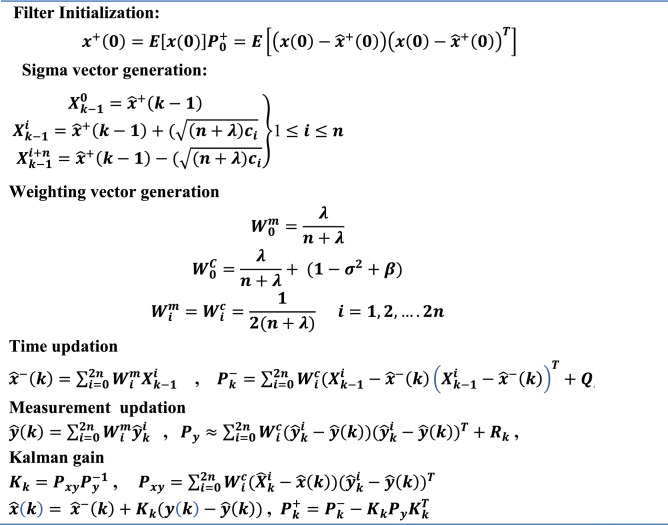
Summary of UKF algorithm.

## Battery modeling

**Figure 3:**
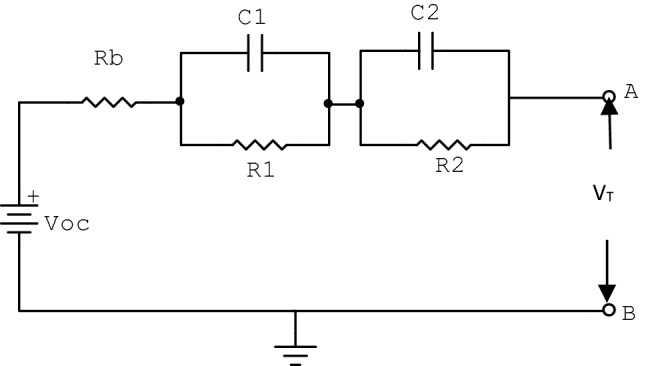
Equivalent circuit model of the battery pack.

Model based fault diagnosis method is implemented using electrochemical properties of a battery. An extension of the venin model is presented which is already applied for various fault diagnosis and state estimation problem. The extended model is used because of the complexity in computation of partial differential equations in electrochemical models. A second order battery model of an additional RC parallel circuit element as shown in Fig. 3 is considered to represent the electrochemical phenomenon of cells. The parameters are interfacial impedance, reactivity distribution of the electrode and the resistance of electron and ion migration. The equivalent circuit consists of a controlled or open circuit voltage source *V*_*oc*_ and change in its value with the SOC, a resistor R_b_ denotes the bulk electrolyte resistance which can vary during the process of charging/ discharging. The constant phase element (C_1_) and resistance (R_1_) makes resistor–capacitor (RC) networks used as model of reactivity capturing model of electrode and the other second RC network combination of R_2_ and C_2_ denote the resistance and capacitance of charge transfer respectively. The current (I) shows charging/discharging current of the system, the performance of a battery pack is greatly affected by the parameters like current, internal resistance and terminal voltage. These parameters are responsible to regulate inconsistency quality, the mode of connection, the variable capacity of cells at different discharge current rate, etc. The resistance–capacitance electrical circuits can be used to model a third order system for battery cells. The each elements of the circuit are the function of SOC and temperature. In the present study the temperature is kept constant, the voltage is varied as a function of SOC and aging dynamics have been kept aside in the model. The significant aspect to be considered is that, the signature fault which may occur in the battery while in operation can be modeled to study behavior of the system under abnormal situations. Effective control of fault estimation also improves the battery life to a large extent. The failure of battery due to overcharging leads to generation of excessive heat due to increase in temperature may cause violent thermal runaways. Moreover, the detrimental copper plating which occurs at the negative electrode of the battery significantly influences the failure of over discharging leading to further thermal runaways. Different types of variation in parameters are noticed during failure of the battery cells due to overcharging and over discharging. It is observed that the increase in bulk resistance (R_b_) is more during overcharging than that of over discharging. Also, the charge transfer resistance (R_1_, R_2_) varies proportionally with both overcharging and over discharging condition. The variation of double layer capacitance (C_1_) and the charge transfer capacitor (C_2_) show a steep increase with over discharging, but the same is very small with gradual dipping in nature is seen in case of overcharging.

**Figure 4:**
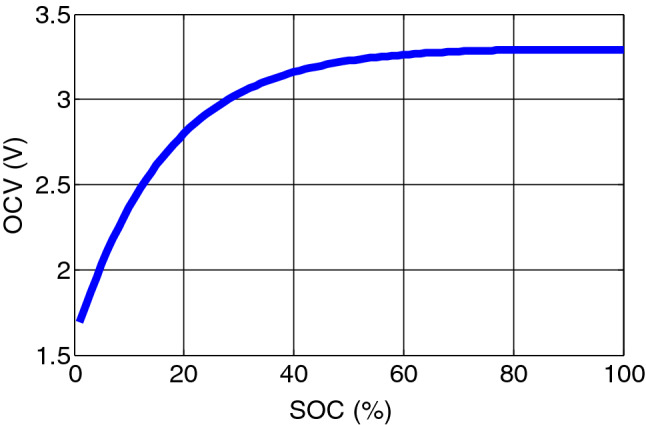
OCV-SOC characteristic for LiFePO_4_ battery cell.

The dynamic equations of the equivalent model of the battery can be represented by

12 $${\dot{V}}_{1}\left(t\right)= -\frac{1}{{R}_{1}{C}_{1}}{V}_{1}\left(t\right)+\frac{1}{{C}_{1}},$$

13 $${\dot{V}}_{2}\left(t\right)= -\frac{1}{{R}_{2}{C}_{2}}{V}_{2}\left(t\right)+\frac{1}{{C}_{2}},$$

14 $${V}_{T}\left(t\right)={V}_{OC}\left(t\right)-{V}_{1}\left(t\right)-{V}_{2}\left(t\right)-I\left(t\right){R}_{b},$$

where, *V*_*T*_, *V*_1_ and *V*_2_ denote the terminal voltage and capacitor voltage across *C*_1_ and *C*_2_ respectively. Open circuit voltage *V*_*oc*_ is a nonlinear function of SOC and described by

15 $${V}_{OC}\left(t\right)=\sum_{k=0}^{m}{C}_{k}{SOC}^{k}\left(t\right),$$

where, coefficients *C*_*k*_, for k = 0,1,2,……..,m are obtained from OCV-SOC characteristic shown in Fig. 4.

The SOC, calculated by the coulomb counting method is given as:

$$\frac{dSOC(t)}{dt}=\frac{\eta {I}_{b}}{{C}_{a}},$$

16 $$or\,\, SOC(t)= SOC(0)+\int\limits_{0}^{\tau}\frac{\eta {I}_{b}(\tau )}{{C}_{a}}d\tau ,$$

where, ${C}_{a}$ is the battery available capacity, and $\upeta$ is the coulomb efficiency that is the function of the current and temperature. $$\upeta =\left\{ {\begin{array}{*{20}l} 1 \hfill & {for\;charging} \hfill \\ {0.95} \hfill & {for\;discharging} \hfill \\ \end{array} } \right.$$.

The model parameter are kept constant neglecting changes occurred due to ageing effect. To simulate with the discrete Kalman filter, the filter model is discretized using Taylor series expansion and neglecting higher terms given as

17 $$V_{1} \left( {k + 1} \right) = \left( {e^{{ - t_{s} /R_{1} C_{1} }} } \right)V_{1} \left( k \right) + R_{1} \left( {1 - ~e^{{ - t_{s} /R_{1} C_{1} }} } \right)I_{b} \left( k \right),$$

18 $$V_{2} \left( {k + 1} \right) = \left( {e^{{ - t_{s} /R_{2} C_{2} }} } \right)V_{1} \left( k \right) + R_{2} \left( {1 - ~e^{{ - t_{s} /R_{2} C_{2} }} } \right)I_{b} \left( k \right),$$

19 $$SOC\left(k+1\right)=SOC\left(k\right)- \frac{{ \eta t}_{s}{I}_{b}\left(k\right)}{{C}_{a}},$$

20 $$\begin{aligned} V_{T} \left( k \right) = & V_{oc} \left( k \right) + V_{1} \left( k \right) + V_{2} \left( k \right) + R_{b} I_{b} \left( k \right) \\ = & C_{7} SOC^{7} \left( k \right) + C_{6} SOC^{6} \left( k \right) + C_{5} SOC^{5} \left( k \right) + C_{4} SOC^{4} \left( k \right) \\ & + C_{3} SOC^{3} \left( k \right) + C_{2} SOC^{2} \left( k \right) + C_{1} SOC\left( k \right) + C_{0} + V_{1} \left( k \right) + V_{2} \left( k \right) + R_{b} I_{b} \left( k \right) \\ = & f\left( {SOC\left( k \right)} \right) + V_{1} \left( k \right) + V_{2} \left( k \right) + R_{b} I_{b} \left( k \right). \\ \end{aligned}$$

These can be expressed as state variable form as

21 $$\left[ {\begin{array}{*{20}c} {SOC\left( {k + 1} \right)} \\ {V_{1} \left( {k + 1} \right)} \\ {V_{2} \left( {k + 1} \right)} \\ \end{array} } \right]~ = ~\left[ {\begin{array}{*{20}c} 1 & 0 & 0 \\ 0 & {\left( {e^{{ - t_{s} /R_{1} C_{1} }} } \right)} & 0 \\ 0 & 0 & {\left( {e^{{ - t_{s} /R_{2} C_{2} }} } \right)} \\ \end{array} } \right]\left[ {\begin{array}{*{20}c} {SOC\left( k \right)} \\ {V_{1} \left( k \right)} \\ {V_{2} \left( k \right)} \\ \end{array} } \right]~ + ~~\left[ {\begin{array}{*{20}c} { - \frac{{~\eta t_{s} }}{{C_{a} }}} \\ {R_{1} \left( {1 - ~e^{{ - t_{s} /R_{1} C_{1} }} } \right)} \\ {R_{2} \left( {1 - ~e^{{ - t_{s} /R_{2} C_{2} }} } \right)} \\ \end{array} } \right]I_{b} \left( k \right),$$

22 $${V}_{T}\left(k\right)=\left[\begin{array}{ccc}f\left(SOC\left(k\right)\right)& 1& 1\end{array}\right]\left[\begin{array}{c}SOC(k)\\ {V}_{1}\left(k\right)\\ {V}_{2}\left(k\right)\end{array}\right]+ \left[{R}_{b}\right]{I}_{b}\left(k\right) .$$

## Simulation result and discussion

A 123 26650 LiFePO_4_ battery (3.3 Volts, 2.5 Ah) cell was used in the experiment. Tables 2 and 3 illustrate the impedance spectroscopy results for the selected circuit parameters variation when the battery cell was under over-charge and over-discharge fault conditions. During over-charge condition battery cell is kept with 120% charge and 100% nominal discharge while during over–discharge condition it is kept in reverse way. In each fault condition spectroscopy measurement for parameter variation of some specific cycles are taken and shown in Tables 2 and 3. Various faults in lithium ion battery cells can be observed by different parameter variation in battery during operation. The paper primarily focused on over-charging (OC) fault, over-discharging (OD) fault and short circuit fault between inter cell power of lithium-ion batteries. The OC condition is achieved by charging the battery to 120% and 100% nominal discharge at a favorable current rate. The variation of system parameters such as R_b_, R_1_, R_2_, C_1_ and C_2_, which significantly contributed in faults during OC and over-discharging (OD) of battery cell parameter variation as seen in the impedance spectroscopy are shown in Tables 2 and 3.

**Table 2:** Variation of system parameters (over-charge).

Cycle	${R}_{b }\,(\Omega )$	${C}_{1}$ (F)	${R}_{2} \,(\Omega )$	${C}_{2}$ (F)	${R}_{1}\, (\Omega )$
1	0.0771	0.0265	0.0156	0.4177	0.0282
5	0.2433	0.00041	0.0369	0.2463	0.0329
10	0.1387	0.00012	0.1429	0.3421	0.0342
12	0.1661	0.0001	0.1734	0.3657	0.0389
15	0.2865	0.0007	0.2134	0.3867	0.04327

**Table 3:** Variation of system parameters (over-discharge).

Cycle	${R}_{b}\,(\Omega )$	${C}_{1}\, (F)$	${R}_{2} \,(\Omega )$	${C}_{2}$ (F)	${R}_{1}\,(\Omega )$
1	0.0771	0.0265	0.0156	0.4177	0.0282
5	0.2433	0.00041	0.0369	0.2463	0.0329
10	0.1387	0.00012	0.1429	0.2567	0.0438
12	0.1661	0.00010	0.1654	0.3569	0.0541
15	0.2865	0.0007	0.1875	0.6541	0.0654

Sinusoidal current as input signal is used as charging or discharging current of the model.

The terminal voltage, state of charge, voltage across *C*_1_ and *C*_2_ in each sampling time is evaluated from the Eqs. (21) and (22).The battery model is run by bank of UKF and EKF to calculate the estimated state of charge, voltage across *C*_1_ and *C*_2_ in each sampling time with healthy and faulty state while the input signal is corrupted with Gaussian white noise with process noise covariance *Q*_*k*_ and measurement noise covariance *R*_*k*_ are taken as Q = 10^–6^ $$\left[\begin{array}{ccc}1& 0& 0\\ 0& 1& 0\\ 0& 0& 1\end{array}\right]$$ and R = 1 × 10^–6^ respectively.

Simulation result deals with performance comparison between UKF and EKF while fault diagnosis of lithium ion battery of electric vehicle. Over-charging, over-discharging and short circuit faults are experimented in battery model and each case for fault diagnosis bank of UKF and EKF are operated. The three states of battery models, those are state of charge, voltage across *C*_1_ and *C*_2_ are estimated and compared to get residual signal in each time step.

The charging current is taken as input signal considered as I = 5sin100πt with initial values of voltage across charge transfer capacitance and double layer capacitances are taken as 0.1 V each. The model is simulated with healthy condition and at 50th sampling instant a fault is injected as overcharge and at 120th sampling instant second fault occurs. As the system is modeled with three state variables as SOC, V_1_ and V_2_, the occurrences of any fault will affect the states of battery model. By comparing the true state and estimated state during healthy and faulty condition is easily detected by residual signal generation.

### Single fault diagnosis

**Figure 5:**
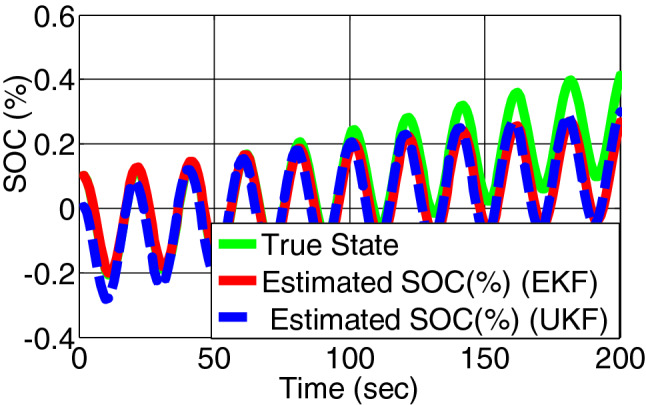
SOC estimation by EKF and UKF on simulated battery model.

**Figure 6:**
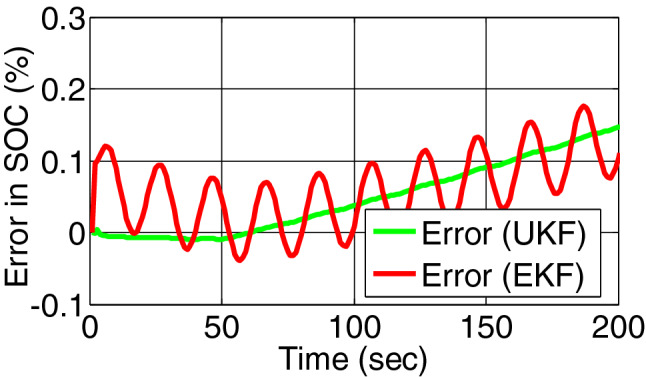
Error/residual evaluation for SOC estimation by EKF and UKF on simulated battery model.

In the proposed battery model is first run healthy condition and at 50th sampling instant a fault is injected as overcharge. Figure 5 represents the true state and estimated state of SOC by EKF and UKF. Figure 6 represents the residual of SOC of both the filter. For both cases the change of residual signal from zero to other value is more appropriate in UKF than EKF.

**Figure 7:**
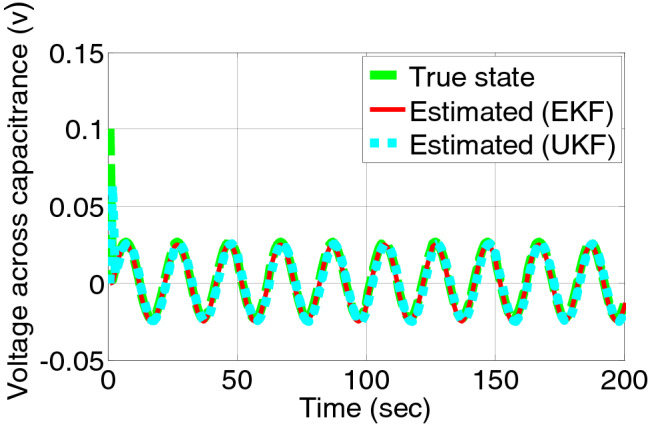
Voltage across charge transfer capacitance estimated by EKF and UKF during charging.

**Figure 8:**
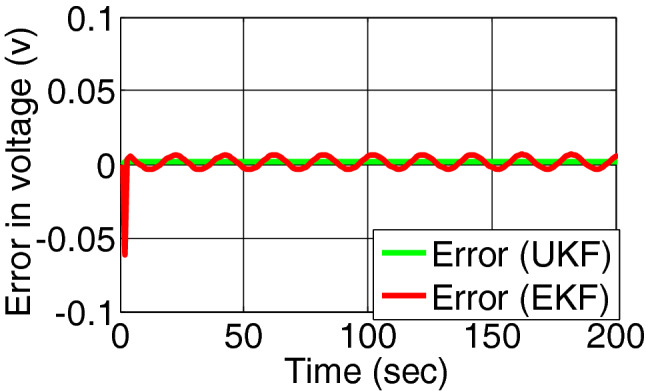
Error/residual evaluation for voltage across charge transfer capacitance estimation by EKF and UKF.

Figures 7 and 8 shows the estimated state of voltage across charge transfer capacitance and the residual signal of both the filter. The residual measurement and state estimation do not reflect the occurrences of fault as overcharge does not affect the voltage across charge transfer capacitances.

**Figure 9:**
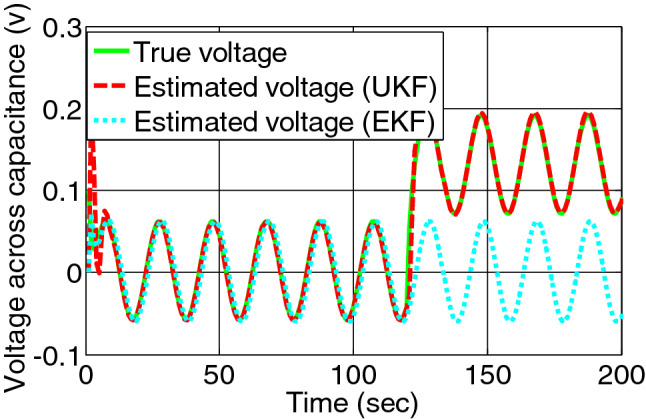
Voltage across charge transfer capacitance estimated by EKF and UKF during over discharging.

**Figure 10:**
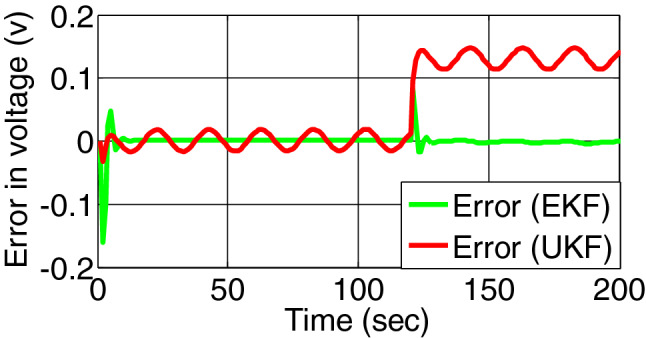
Error/residual evaluation for Voltage across charge transfer capacitance estimation by EKF and UKF during discharging.

When over discharging fault occurs at 120th second, the true voltage and estimated voltage across charge transfer capacitance by both filters are represented by Fig. 9. The residual signal of both the filters is shown in Fig. 10.

The shift of residual for second fault is clear for UKF than EKF. Under this condition the residual for SOC is unaffected showing zero.

### Multiple fault diagnosis

**Figure 11:**
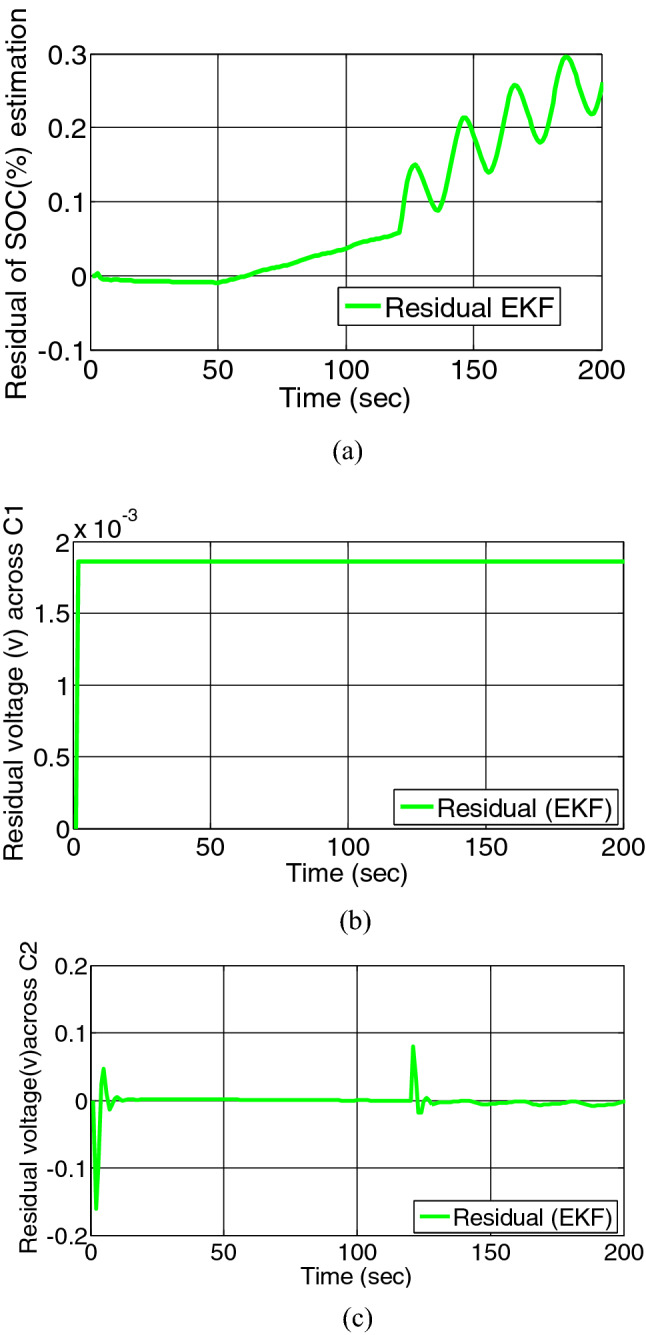
(**a**) Residual estimation of SOC, (**b**) residual estimation of voltage across charge transfer capacitance. (**c**) Residual estimation of voltage across double layer capacitance by EKF.

**Figure 12:**
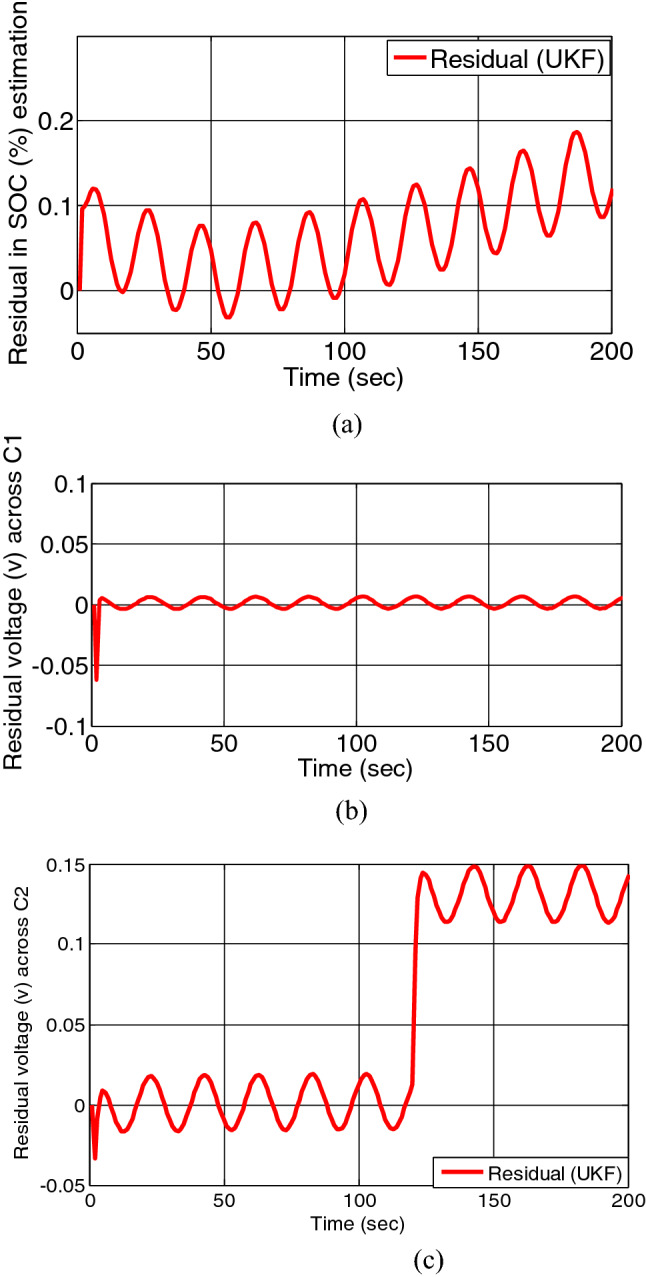
(**a**) Residual estimation of SOC, (**b**) residual estimation of voltage across charge transfer capacitance, (**c**) residual estimation of voltage across double layer capacitance by UKF.

When overcharging fault at 50th s and short circuit fault across charge transfer resistance at 120th s both fault occurs on battery model simultaneously, the residual of SOC and voltage across charge transfer capacitance and double layer capacitance are depicted in Figs. 11a–c and 12a–c.

## Conclusion

In the present study, a discrete non-linear mathematical model of lithium-ion battery has been developed for multiple fault detection using two non-linear Kalman filters. The performance comparison using bank of UKF and EKF for single and simultaneous occurrences of multiple fault diagnosis such as over-charge, over-discharge and short circuit fault between inter cell power in lithium-ion battery has been carried out. In the proposed fault diagnosis scheme both (UKF and EKF) bank of filters are employed separately on lithium-ion battery model during normal and faulty situation so that the filters output and measured output are compared to generate residual signals. It has been shown from the simulation results of statistical test that residual signal under no fault indicates zero mean signal within threshold value whereas it exceeds the threshold value with non-zero mean signal during faulty condition. The comparison result for both the filter (UKF and EKF) from simulation study proves that UKF model exhibits better and quicker response than that of EKF for multiple fault diagnosis of lithium-ion battery model.

## Data Availability

All data generated or analysed during this study are included in this published article.
